# Supplementary material for: Hippocampal representations switch from errors to predictions during acquisition of predictive associations
Source: Nat Commun. 2022 Jun 8;13:3294. doi: 10.1038/s41467-022-31040-w (PMC9178037; doi:10.1038/s41467-022-31040-w)
Supplement: Supplementary file 1 — Supplementary Information [file 41467_2022_31040_MOESM1_ESM.pdf]

Hippocampal representations switch from errors to predictions during acquisition of predictive associations

Fraser Aitken[1,2], Peter Kok[1*]

[1]Wellcome Centre for Human Neuroimaging, UCL Queen Square Institute of Neurology, University College London, 12 Queen Square, London WC1N 3AR, UK
[2]School of Biomedical Engineering and Imaging Sciences, King's College London, St Thomas' Hospital, London SE1 7EH, UK

[*]**Corresponding author**
Wellcome Centre for Human Neuroimaging
UCL Queen Square Institute of Neurology
12 Queen Square, London WC1N 3AR, UK
Phone: +44 (0)20 3448 4341
E-mail: p.kok@ucl.ac.uk

Supplementary Information

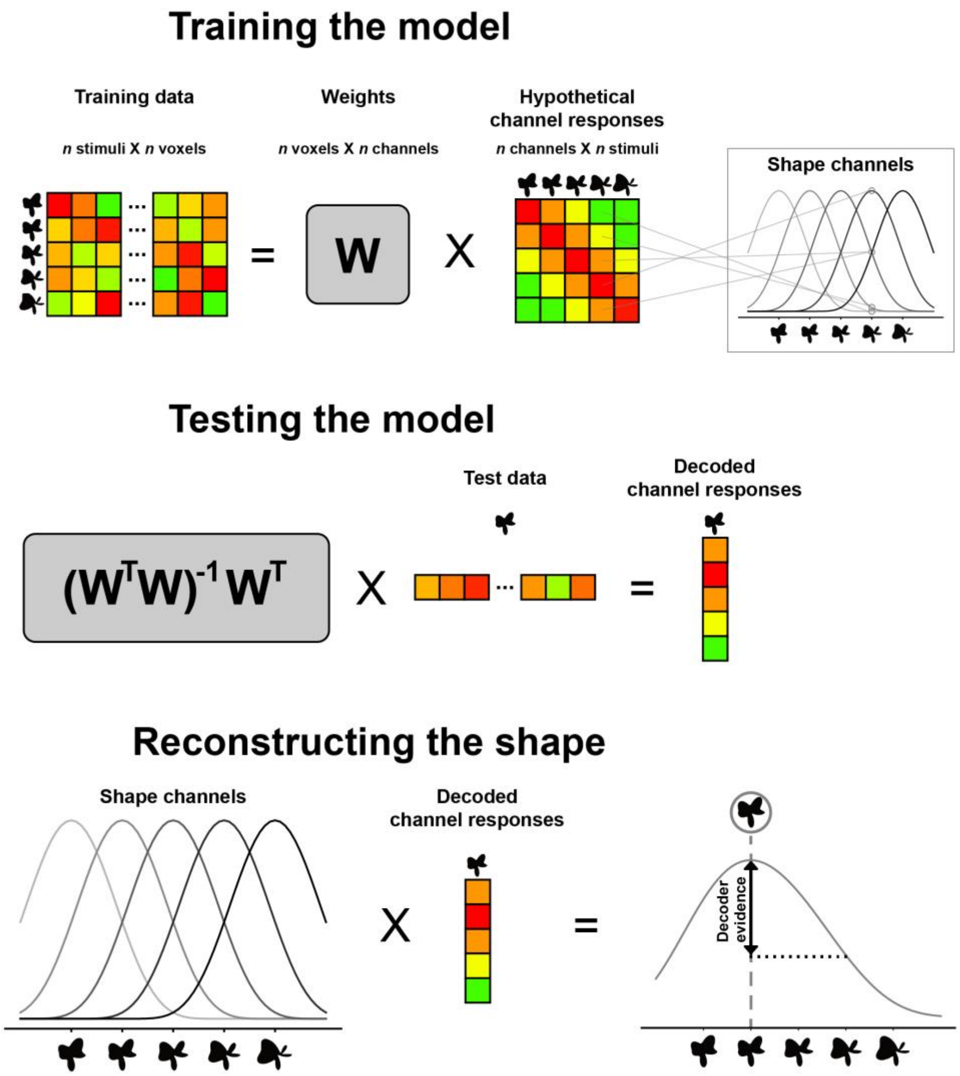

Supplementary Fig. 1. Illustration of shape decoding analysis. In order to probe neural shape representations, a forward modelling approach was used to decode the shapes from the patterns of BOLD activity in each ROI. In the first stage of the analysis, the parameter estimates obtained from the two shape-only runs were used to estimate the weights on the five hypothetical channels separately for each voxel, using linear regression. This constituted training the model. The second stage, testing the model, consisted of estimating the channel outputs associated with the pattern of activity across voxels evoked by each trial in the prediction runs, again using linear regression. These estimated channel outputs were used to compute a weighted average of the five basis functions, reflecting a neural shape tuning curve. Note that, during the main experiment (i.e., the prediction runs), only shapes 2 and 4 were presented. Decoding performance was quantified by subtracting the amplitude of the shape tuning curve at the presented shape (e.g., shape 2) from the amplitude at the non-presented shape (shape 4).

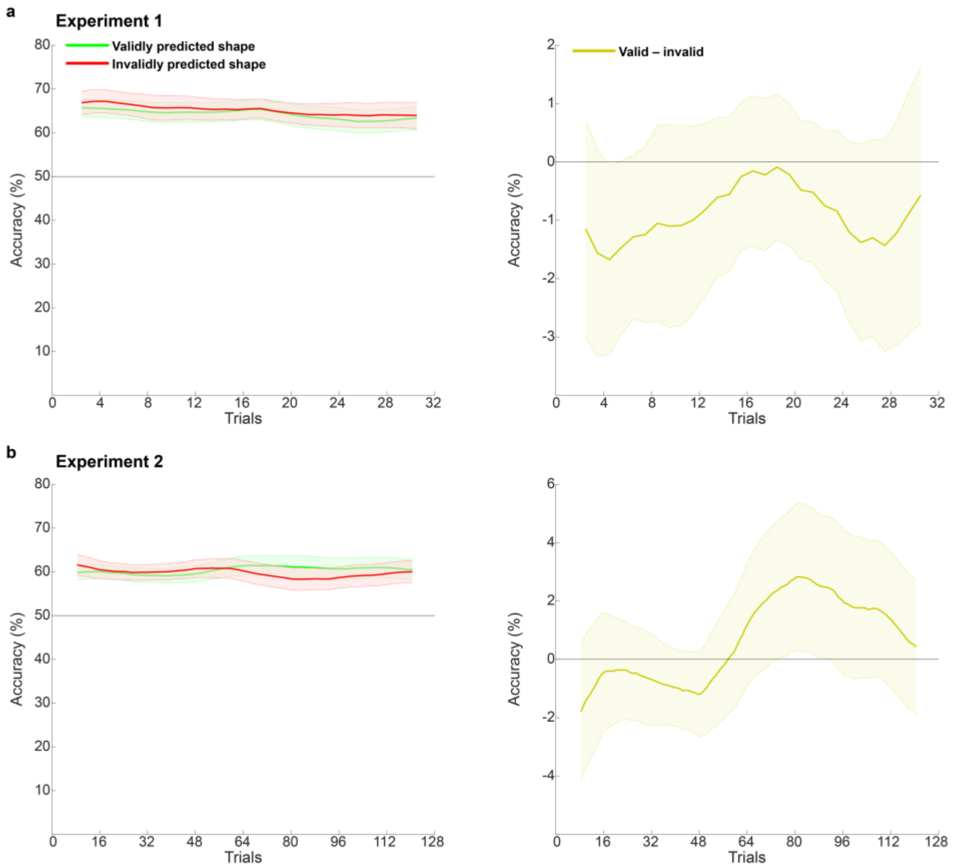

Supplementary Fig. 2. Behavioural performance over trials. a Task accuracy on trials with validly predicted (green) and invalidly predicted (red) shapes in Experiment 1 (left panel). Difference in accuracy between valid and invalid trials (yellow, right panel). **b** Task accuracy on trials with validly predicted (green) and invalidly predicted (red) shapes in Experiment 2 (left panel). Difference in accuracy between valid and invalid trials (yellow, right panel). N = 24 participants in all panels. Shaded regions indicate SEM.

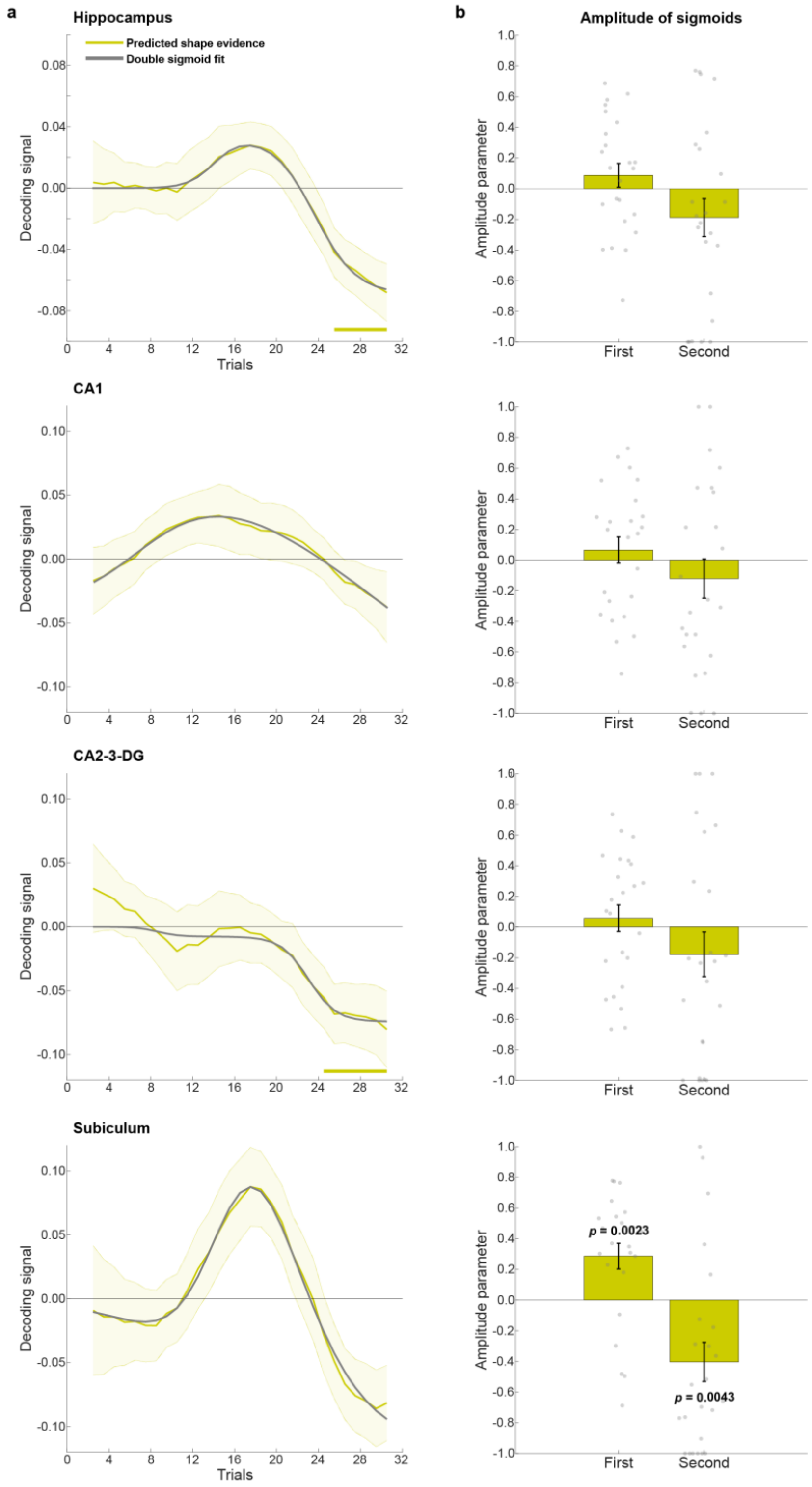

Supplementary Fig. 3. Quantification of hippocampal effects in Experiment 1 with two sigmoids. a Double sigmoid learning curve fit to predicted shape decoding in hippocampus and its subfields. Horizontal lines indicate significant clusters. N = 24 participants. Shaded regions indicate SEM. **b** Amplitude parameters of the two sigmoids making up the fitted curve. N = 24 participants. Error bars indicate SEM. Dots indicate individual participants. P values reflect two-sided one-sample *t*-tests (df = 23) against zero. Source data are provided as a Source Data file.

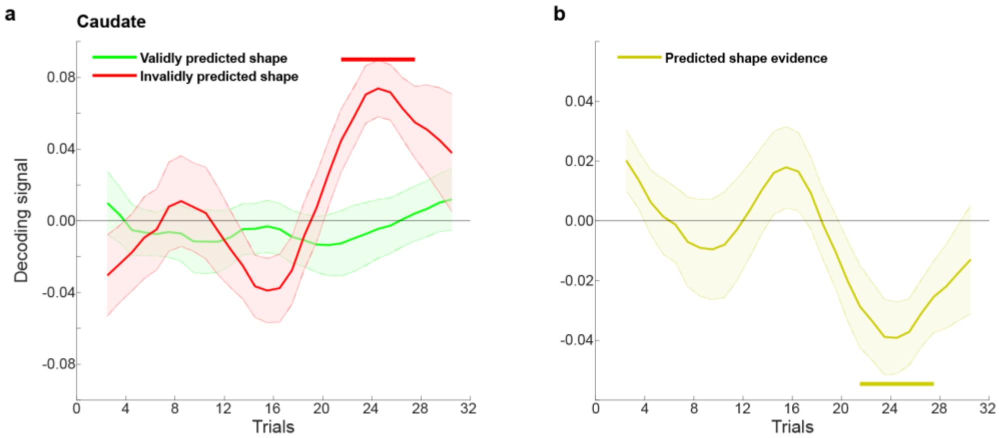

Supplementary Fig. 4. Experiment 1 shape decoding over trials in the caudate nucleus. a Decoding evidence for validly (green) and invalidly (red) predicted shapes in the caudate. **b** Decoding evidence for predicted (valid – invalid) shapes in the caudate. Horizontal lines indicate significant clusters. N = 24 participants, shaded regions indicate SEM.

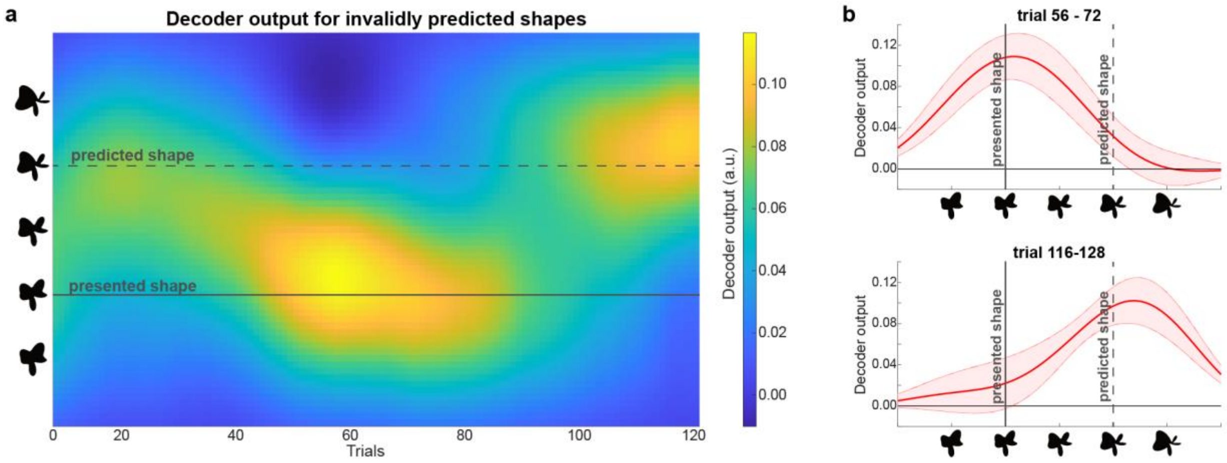

Supplementary Fig. 5. Evolution of shape decoder output on invalid trials in the hippocampus. a Shape decoder output for invalidly predicted shapes, over trials (x-Axis), across the full range of possible shapes as presented in the shape-only runs (y-Axis). See Supplementary Fig. 1 and Methods for more details on the shape decoding algorithm. Note that these are the same data underlying the red time course in Fig. 5a, which consists of the subtraction of evidence for the presented shape (here, shape 2) minus the non-presented shape (here, shape 4, in this case the predicted shape). **b** Two vertical slices from panel A; decoder output for invalid shapes halfway through the blocks (trials 56-72, top panel) and at the end of the blocks (trials 116-128, bottom panel). Halfway through, evidence for the presented shape (shape 2) was stronger than for the shapes 1 (t_{23} = 2.22, p = 0.036), 4 (the predicted shape; t_{23} = 2.59, p = 0.016), and 5 (t_{23} = 3.17, p = 0.0043), but not for shape 3 (t_{23} = 1.06, p = 0.30). At the end of the blocks, evidence for the predicted shape (shape 4) was stronger than for shapes 1 (t_{23} = 2.60, p = 0.016) and 2 (the presented shape; t_{23} = 2.07, p = 0.050), but not significantly different from evidence for shapes 3 (t_{23} = 1.78, p = 0.088) and 5 (t_{23} = 0.92, p = 0.37). N = 24 participants, shaded regions indicate SEM.

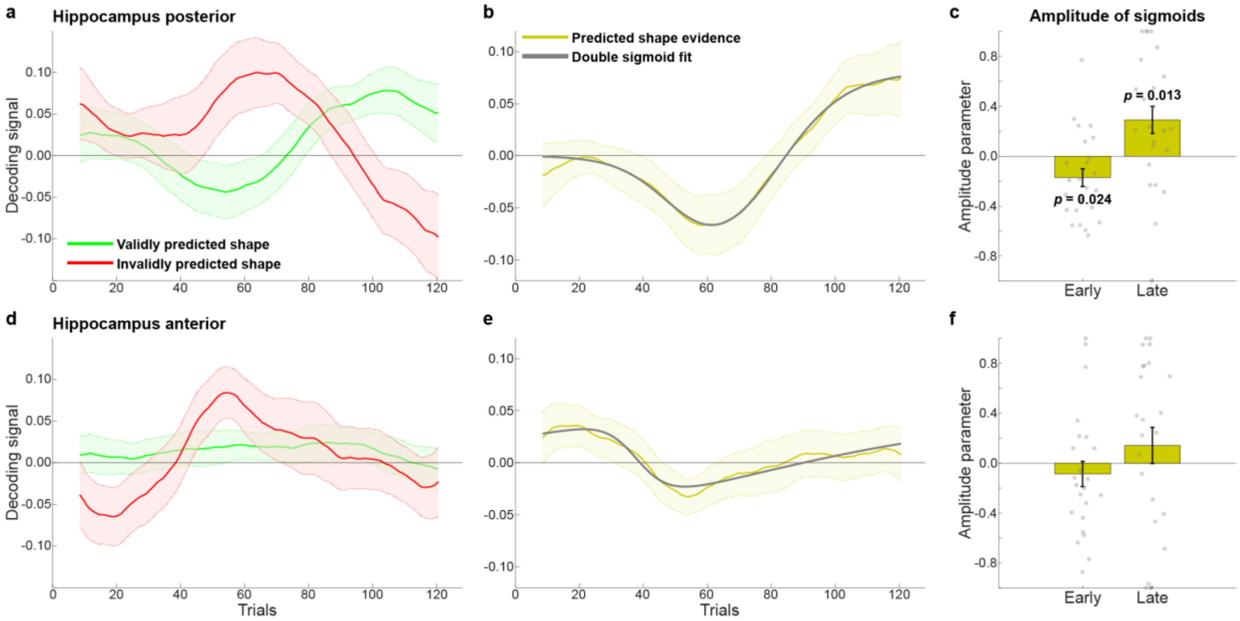

Supplementary Fig. 6. Experiment 2 shape decoding over trials in posterior and anterior hippocampus. a Decoding evidence for validly (green) and invalidly (red) predicted shapes in posterior hippocampus. **b** Decoding evidence for predicted (valid – invalid) shapes in posterior hippocampus (yellow) with double sigmoid fit (gray). **c** Amplitude parameters of early (midpoint between trials 1 and 64) and late (midpoint between trials 65 and 128) sigmoid curves in posterior hippocampus. **d** Decoding evidence for validly (green) and invalidly (red) predicted shapes in anterior hippocampus. **e** Decoding evidence for predicted (valid – invalid) shapes in anterior hippocampus (yellow) with double sigmoid fit (gray). **f** Amplitude parameters of early (midpoint between trials 1 and 64) and late (midpoint between trials 65 and 128) sigmoid curves in anterior hippocampus. N = 24 participants in all panels. Shaded regions and error bars indicate SEM. Dots indicate individual participants. P values reflect two-sided one-sample *t*-tests (df = 23) against zero. Source data are provided as a Source Data file.

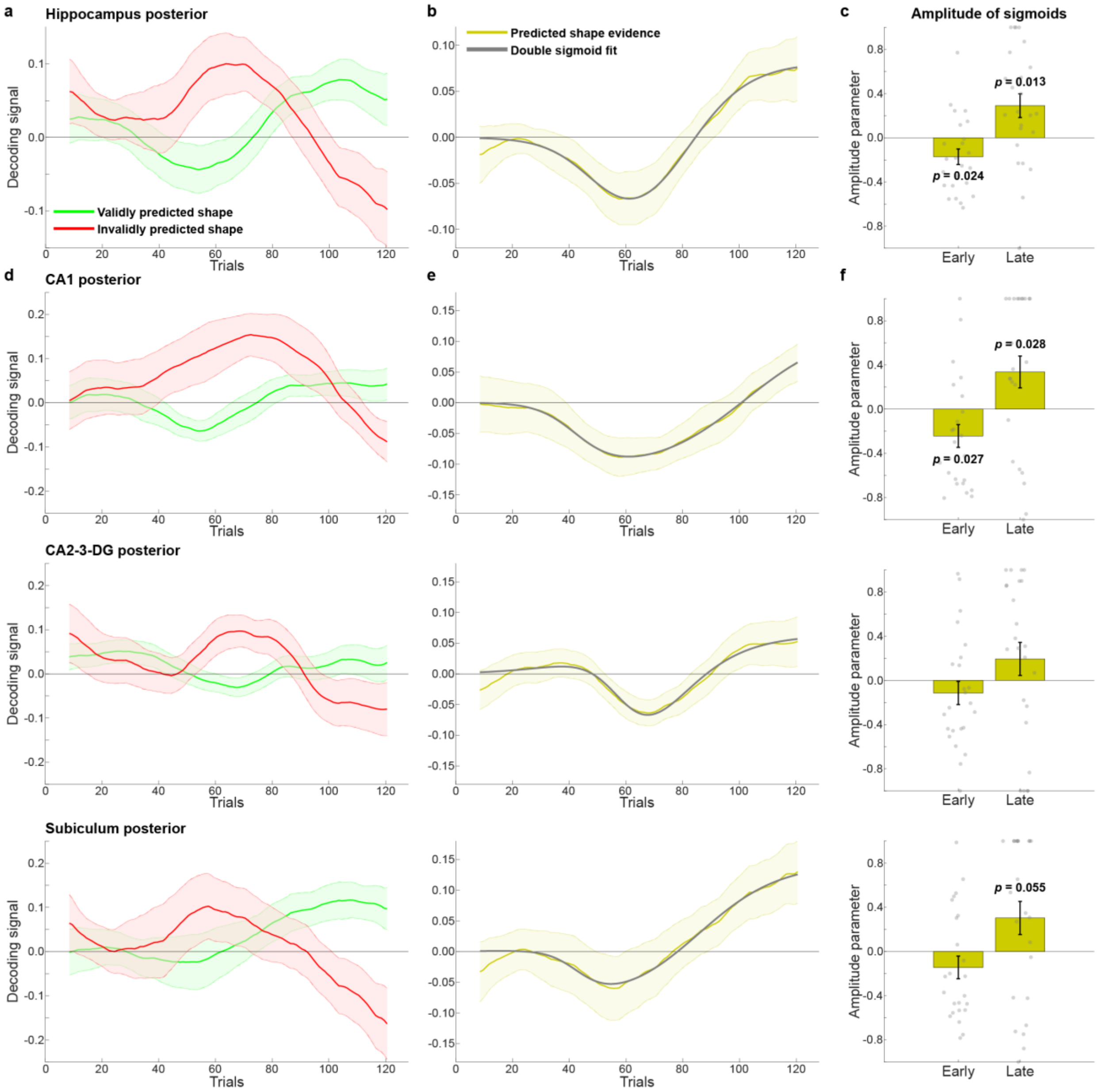

Supplementary Fig. 7. Experiment 2 shape decoding over trials in posterior hippocampus. a Decoding evidence for validly (green) and invalidly (red) predicted shapes in posterior hippocampus. **b** Decoding evidence for predicted (valid – invalid) shapes in posterior hippocampus (yellow) with double sigmoid fit (gray). **c** Amplitude parameters of early (midpoint between trials 1 and 64) and late (midpoint between trials 65 and 128) sigmoid curves in hippocampus. **d** Decoding evidence for validly (green) and invalidly (red) predicted shapes in posterior hippocampal subfields. **e** Decoding evidence for predicted (valid – invalid) shapes in posterior hippocampal subfields (yellow) with double sigmoid fit (gray). **f** Amplitude parameters of early (midpoint between trials 1 and 64) and late (midpoint between trials 65 and 128) sigmoid curves in posterior hippocampal subfields. N = 24 participants in all panels. Shaded regions and error bars indicate SEM. Dots indicate individual participants. P values reflect two-sided one-sample *t*-tests (df = 23) against zero. Source data are provided as a Source Data file.

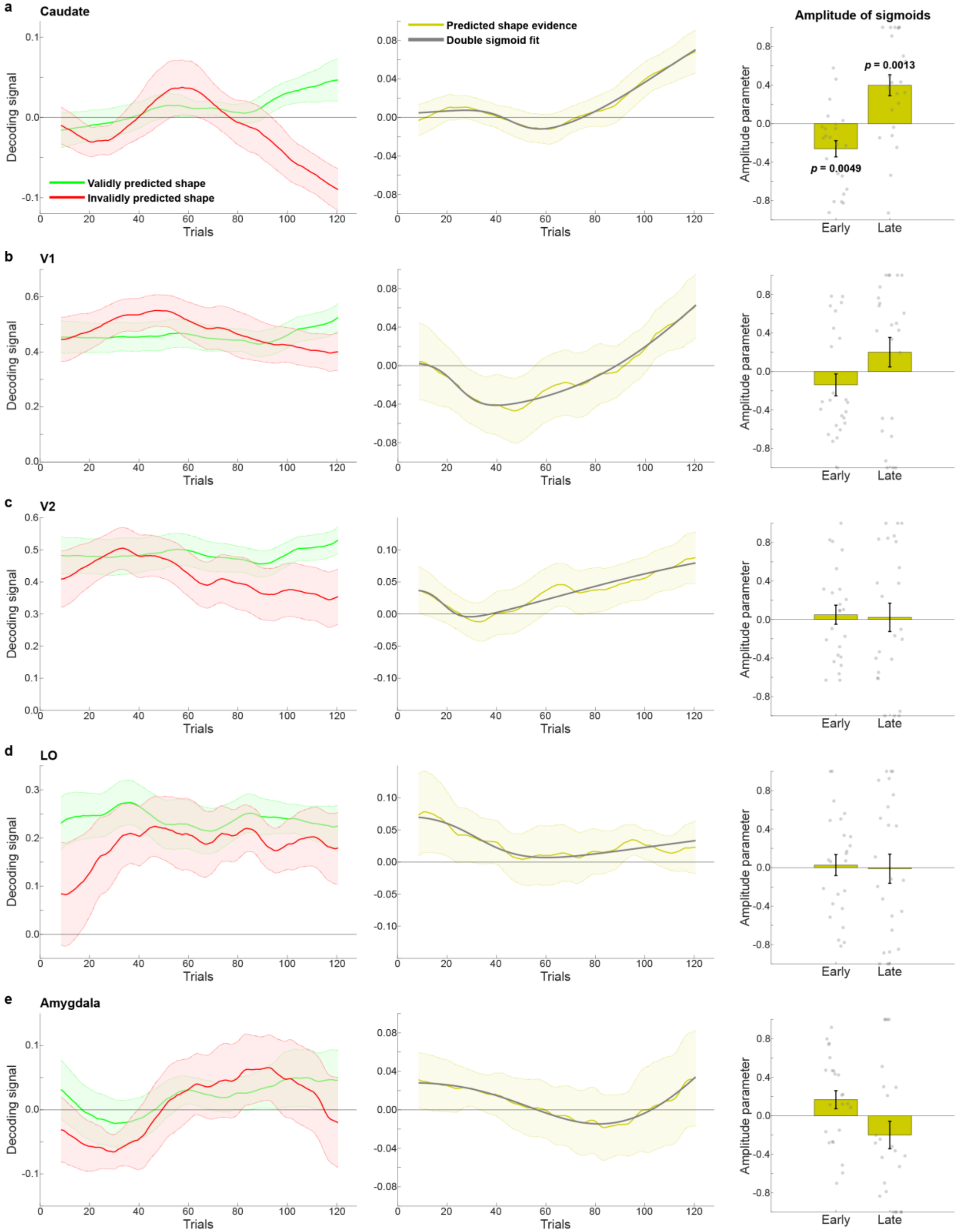

Supplementary Fig. 8. Experiment 2 shape decoding over trials in additional regions. Decoding evidence for validly (green) and invalidly (red) predicted shapes (left panel), decoding evidence for predicted (valid – invalid) shapes (middle panel), and amplitude parameters of early (midpoint between trials 1 and 64) and late (midpoint between

trials 65 and 128) sigmoid curves (right panel) in **a** the caudate nucleus, **b** V1, **c** V2, **d** Lateral occipital cortex, **e** Amygdala. N = 24 participants in all panels. Shaded regions and error bars indicate SEM. Dots indicate individual participants. P values reflect two-sided one-sample *t*-tests (df = 23) against zero. Source data are provided as a Source Data file.
