# Supplementary material for: Hippocampal representations switch from errors to predictions during acquisition of predictive associations
Source: Nat Commun. 2022 Jun 8;13:3294. doi: 10.1038/s41467-022-31040-w (PMC9178037; doi:10.1038/s41467-022-31040-w)
Supplement: Supplementary file 3 — Reporting Summary [file 41467_2022_31040_MOESM3_ESM.pdf]

nature portfolio

Corresponding author(s): Peter Kok

Last updated by author(s): Apr 7, 2022

Reporting Summary

Nature Portfolio wishes to improve the reproducibility of the work that we publish. This form provides structure for consistency and transparency in reporting. For further information on Nature Portfolio policies, see our Editorial Policies and the Editorial Policy Checklist.

Statistics

For all statistical analyses, confirm that the following items are present in the figure legend, table legend, main text, or Methods section.

n/a	Confirmed	
☐	☒	The exact sample size (*n*) for each experimental group/condition, given as a discrete number and unit of measurement
☐	☒	A statement on whether measurements were taken from distinct samples or whether the same sample was measured repeatedly
☐	☒	The statistical test(s) used AND whether they are one- or two-sided<br>*Only common tests should be described solely by name; describe more complex techniques in the Methods section.*
☒	☐	A description of all covariates tested
☐	☒	A description of any assumptions or corrections, such as tests of normality and adjustment for multiple comparisons
☐	☒	A full description of the statistical parameters including central tendency (e.g. means) or other basic estimates (e.g. regression coefficient) AND variation (e.g. standard deviation) or associated estimates of uncertainty (e.g. confidence intervals)
☐	☒	For null hypothesis testing, the test statistic (e.g. *F*, *t*, *r*) with confidence intervals, effect sizes, degrees of freedom and *P* value noted<br>*Give P values as exact values whenever suitable.*
☒	☐	For Bayesian analysis, information on the choice of priors and Markov chain Monte Carlo settings
☒	☐	For hierarchical and complex designs, identification of the appropriate level for tests and full reporting of outcomes
☐	☒	Estimates of effect sizes (e.g. Cohen's *d*, Pearson's *r*), indicating how they were calculated

Our web collection on statistics for biologists contains articles on many of the points above.

Software and code

Policy information about availability of computer code

Data collection	The auditory and visual stimuli were generated and presented using the Psychtoolbox (version 3) in conjunction with Matlab (version 9).
Data analysis	Behavioural and fMRI data were analysed using Matlab (version 9). Preprocessing of fMRI data was done using SPM12, decoding analyses and statistical tests were performed using custom scripts. Anatomical segmentation was performed using Freesurfer (version 5). All analysis scripts have been made publicly available on the OSF framework: https://osf.io/48xjf/

For manuscripts utilizing custom algorithms or software that are central to the research but not yet described in published literature, software must be made available to editors and reviewers. We strongly encourage code deposition in a community repository (e.g. GitHub). See the Nature Portfolio guidelines for submitting code & software for further information.

Data

Policy information about availability of data

All manuscripts must include a data availability statement. This statement should provide the following information, where applicable:

- Accession codes, unique identifiers, or web links for publicly available datasets
- A description of any restrictions on data availability
- For clinical datasets or third party data, please ensure that the statement adheres to our policy

All region-specific fMRI time course data are available on the OSF platform (DOI 10.17605/OSF.IO/48XJF).

Field-specific reporting

Please select the one below that is the best fit for your research. If you are not sure, read the appropriate sections before making your selection.

☒ Life sciences ☐ Behavioural & social sciences ☐ Ecological, evolutionary & environmental sciences

For a reference copy of the document with all sections, see nature.com/documents/nr-reporting-summary-flat.pdf

Life sciences study design

All studies must disclose on these points even when the disclosure is negative.

Sample size	Both fMRI experiments had a sample size of N=24, based on the sample size of previous fMRI studies using similar decoding analyses of hippocampal activity patterns (Kok & Turk-Browne 2018; Kok et al 2020).
Data exclusions	In Experiment 1, five participants were excluded due to our strict head motion criteria (five or more movements larger than 1.5 mm in any direction between successive functional volumes). In Experiment 2, two participants were excluded for not performing the task above chance, and three due to excessive head motion.
Replication	This study consists of two fMRI experiments; Experiment 2 replicates and extends the findings of Experiment 1. We do not offer a straight up replication, future research should aim to replicate and extend our findings.
Randomization	This study employs a within-subject design, therefore we recruited one group of participants per experiment, and data in all experimental conditions were collected in all participants.
Blinding	Blinding does not apply, since we employed a within-subject design.

Reporting for specific materials, systems and methods

We require information from authors about some types of materials, experimental systems and methods used in many studies. Here, indicate whether each material, system or method listed is relevant to your study. If you are not sure if a list item applies to your research, read the appropriate section before selecting a response.

Materials & experimental systems

n/a	Involved in the study	
☒	☐	Antibodies
☒	☐	Eukaryotic cell lines
☒	☐	Palaeontology and archaeology
☒	☐	Animals and other organisms
☐	☒	Human research participants
☒	☐	Clinical data
☒	☐	Dual use research of concern

Methods

n/a	Involved in the study	
☒	☐	ChIP-seq
☒	☐	Flow cytometry
☐	☒	MRI-based neuroimaging

Human research participants

Policy information about studies involving human research participants

Population characteristics	For both experiments we recruited healthy, right-handed, MR-compatible participants with normal or corrected-to-normal vision. In Experiment 1, the final sample consisted of 24 participants (12 female; age 25.6 ± 7.2, mean ± SD). In Experiment 2, the final sample consisted of 24 participants (19 female; age 26.2 ± 7.0, mean ± SD).
Recruitment	Participants were recruited through the local UCL SONA participant database. This may have led to self-recruitment biases and to a bias towards the student population, but these are unlikely to have affected the low-level neural signals studied here.
Ethics oversight	All participants provided informed consent through a protocol reviewed by the University College London (UCL) Research Ethics Committee.

Note that full information on the approval of the study protocol must also be provided in the manuscript.

Magnetic resonance imaging

Experimental design

Design type	Event-related
Design specifications	All participants took part in one fMRI scanning session, consisting of 6 runs (~12 min per run) of functional data acquisition, plus anatomical scans. Runs 1 and 6 consisted of two blocks of 60 trials each. In Experiment 1, runs 2-5 consisted of four blocks of 32 trials. In Experiment 2, runs 2-5 consisted of two blocks of 64 trials. Each trial lasted 2.1 second, and the intertrial interval was 2-5s.
Behavioral performance measures	Accuracy and reaction time were collected. Average accuracy was above the 50% chance rate for all participants in both experiments, as reported in the manuscript, which demonstrated that participants were performing the task as expected.

Acquisition

Imaging type(s)	functional, structural
Field strength	3T
Sequence & imaging parameters	Functional images for both experiments were acquired using a T2*-weighted multiband echo-planar imaging sequence (TR = 1000 ms; TE = 33.0 ms; 60 transverse slices; voxel size = 1.5 × 1.5 × 1.5 mm; flip angle = 55°, multiband factor = 6). Field map data were acquired using a Siemens Field Map sequence (TR = 1020.0ms; short TE = 10.00ms; long TE = 12.46ms; voxel size = 3.0 × 3.0 × 2.0 mm, 64 transverse slices, flip angle = 90°). Anatomical images were acquired using a T1-weighted Magnetisation Prepared Rapid Gradient Echo (MPRAGE), using a Generalized Auto calibrating Partially Parallel Acquisition (GRAPPA) factor of 2 (TR = 2530 ms; TE = 3.34ms; 176 sagittal slices; voxel size = 1.0 × 1.0 × 1.0 mm; flip angle = 7°). To enable hippocampal segmentation, a T2-weighted turbo spin-echo (TSE) image (TR = 12650 ms; TE = 45 ms; voxel size = 0.4 × 0.4 × 1.5 mm; 54 coronal slices perpendicular to the long axis of the hippocampus; flip angle = 122°) was acquired.
Area of acquisition	This functional sequence produced a partial volume for each participant, which covered the occipital and temporal lobes, including and parallel to the hippocampus.

Diffusion MRI ☐ Used ☒ Not used

Preprocessing

Preprocessing software	SPM12
Normalization	All ROIs were defined in subjects' native space, so no normalisation was performed.
Normalization template	All ROIs were defined in subjects' native space, so no normalisation was performed.
Noise and artifact removal	For each run, the remaining functional images were spatially realigned to correct for head motion, and simultaneously supplied to B0 unwarping, using SPM's realign and unwarp function. The functional data were temporally high-pass filtered with a 128s period cut-off. No spatial smoothing was applied, and all analyses were performed in participants' native space.
Volume censoring	The first six volumes of each functional run were discarded to allow T1 equilibration. No other volume censoring was performed.

Statistical modeling & inference

Model type and settings	Single trial evoked responses were estimated using mass univariate GLMs. Single trial betas were submitted to multivoxel decoding analyses, as specified in the manuscript.
Effect(s) tested	Initially, in Experiment 1, in a fully assumption-free analysis, we performed cluster-based permutation tests103 on the time courses, to test whether the decoding signals differed significantly from zero at any timepoint. Subsequently, the obtained time courses of decoding evidence for the predicted shapes were quantified by fitting sigmoid curves to them. The amplitude parameter of the sygmoids was submitted to simple t-tests to test whether learning curves significantly deviating from zero.

Specify type of analysis: ☐ Whole brain ☒ ROI-based ☐ Both

Anatomical location(s)	The hippocampus and its subfields, CA1, CA2-3-DG, and the subiculum, were defined based on the structural T2 and T1 images using the automatic segmentation of hippocampal subfields (ASHS) machine learning toolbox, in conjunction with a database of manual medial temporal lobe (MTL) segmentations from a separate set of 51 participants. Additionally, a caudate region of interest (ROI), as well as visual cortex ROIs for our informational connectivity analysis – V1, V2 lateral occipital cortex (LO) – were automatically defined in each participant's T1-weighted anatomical scan using FreeSurfer.
Statistic type for inference (See Eklund et al. 2016)	Decoding yielded a summary statistic per ROI, so no voxel-wise tests of cluster corrected were performed.

Correction

We had an a-priori hypothesis on the hippocampus as a whole, which we followed up by investigating its subfields.

Models & analysis

n/a	Involved in the study
☐	☒ Functional and/or effective connectivity
☐	☐ Graph analysis
☐	☐ Multivariate modeling or predictive analysis

Functional and/or effective connectivity

In an exploratory analysis, we investigated whether functional connectivity between regions (specifically, between the posterior subiculum and EC, V1, V2, and LO) changed over trials in Experiment 2. Specifically, the Pearson correlation in decoding evidence over trials between two regions was calculated.

Graph analysis

Report the dependent variable and connectivity measure, specifying weighted graph or binarized graph, subject- or group-level, and the global and/or node summaries used (e.g. clustering coefficient, efficiency, etc.).

Multivariate modeling and predictive analysis

Specify independent variables, features extraction and dimension reduction, model, training and evaluation metrics.
